# Roles of sirtuins in asthma

**DOI:** 10.1186/s12931-022-02175-7

**Published:** 2022-09-18

**Authors:** Yahui Liu, Guochao Shi

**Affiliations:** 1 Department of Respiratory and Critical Care Medicine, Ruijin Hospital, Shanghai Jiao Tong University School of Medicine, Shanghai, People’s Republic of China; 2 Institute of Respiratory Diseases, Ruijin Hospital, Shanghai Jiao Tong University School of Medicine, Shanghai, People’s Republic of China; 3 Shanghai Key Laboratory of Emergency Prevention, Diagnosis and Treatment of Respiratory Infectious Diseases, Ruijin Hospital, Shanghai Jiao Tong University School of Medicine, Shanghai, People’s Republic of China

**Keywords:** Sirtuins, Asthma, SIRT1, Modulators

## Abstract

Sirtuins are nicotinamide adenine dinucleotide (NAD^+^)-dependent lysine deacylases and deacetylases that participate in a variety of cellular processes, including transcriptional activity, energy metabolism, DNA damage response, inflammation, apoptosis, autophagy, and oxidative stress. As a result, sirtuins are linked to multiple pathophysiological processes, such as cardiovascular diseases, metabolic diseases, autoimmune diseases, infectious diseases, and respiratory diseases. Asthma is the most common respiratory disease, which is characterized by airway inflammation and airway remodeling. Accumulating evidence has indicated that sirtuins are involved in the pathogenesis of asthma. Furthermore, some studies have suggested that sirtuin modulators are potential agents for the treatment of asthma via alteration of the expression or activity of sirtuins. In this review, we illustrate the role of sirtuins in asthma, discuss related molecular mechanisms, and evaluate the sirtuins-targeted therapy for asthma.

## Introduction

Asthma is one of the most common respiratory diseases, characterized by variable respiratory symptoms and airflow limitation. According to a national survey, 7.7% of people in the United States have asthma [1]. The prevalence of asthma among adults in China is 4.2%, with an estimated 45.7 million asthma patients [2]. Beyond inhaled corticosteroids and long-acting β_2_ agonists, other add-on therapies are also considered, such as leukotriene receptor antagonists, long-acting muscarinic antagonists (for patients aged ≥ 12 years), systemic corticosteroids, and biologics. Despite so many options for treating asthma, many people with asthma remain poorly controlled [2]. Asthma causes a heavy disease burden to society. Increasing numbers of people are engaged in research on asthma to clarify the pathogenesis of asthma and better treat it. However, many questions surrounding asthma require further study.

The sirtuin (SIRT) family consists of seven members (SIRT1–SIRT7), which share homology with the yeast silent information regulator 2 (Sir2) protein [3]. SIRTs have received a lot of attention over the past 2 decades. Original studies have indicated that SIRTs, as class III lysine deacetylases (KDACs), are widely involved in regulating aging and lifespan in humans. Subsequent studies have shown that SIRTs are involved in various cellular functions and physiological processes via their deacetylase and mono-adenosine diphosphate (ADP)-ribosyltransferase activities, such as transcriptional activity, energy metabolism, DNA damage response, inflammation, apoptosis, autophagy, and oxidative stress [4, 5]. Many findings on SIRTs have established their function in the pathogenesis of asthma. Here, to provide a novel therapeutic strategy for treatment, we reviewed the literature to discuss the roles and related molecular pathways of each member of the SIRT family in asthma.

## Pathogenesis of asthma

Asthma is a highly heterogeneous disease characterized by airway inflammation and airway remodeling. Huge progress has been made in our understanding of the pathogenesis and heterogeneity of asthma. According to sputum cytology classification, airway inflammation of patients with asthma can be classified into four types: eosinophilic, neutrophilic, mixed complex, and paucigranulocytic inflammation [6]. Later, researchers identified two distinct molecular phenotypes of asthma, mainly defined by the degree of T helper 2 (Th2) inflammation, “Th2-high” and “Th2-low” asthma [7]. Th2-high asthma is the most common and most well-studied phenotype of asthma, which is always accompanied by eosinophilic inflammation and anaphylaxis [8]. Airway epithelial cells secrete interleukin 33 (IL-33), IL-25,  and thymic stromal lymphopoietin after being stimulated by allergic sensitization. Under the complex effects of dendritic cells (DCs) and the effects of these cytokines listed above, Th2 cells proliferate and release a large amount of Th2 cytokines, including IL-4, IL-5, and IL-13. IL-4 can act on T and B cells and induce Th2 differentiation and immunoglobulin E (IgE) class-switching, thus playing a key role in asthma. IL-5 is an obligate cytokine regulating the differentiation and function of eosinophils and involved in remodeling and wound healing. IL-13 participates in multiple aspects of asthma, including activation, recruitment, and survival of eosinophils, tissue remodeling, and fibrosis. Some studies have described Th2-low asthma, but it is poorly understood compared with Th2-high asthma [9].

Airway remodeling consists of a series of structural changes in tissues of the airway, including epithelial damage, goblet cell hyperplasia, angiogenesis, reticular basement membrane thickening, fibrosis, and smooth muscle hyperplasia. These alterations in the airways can eventually have a detrimental effect on lung function and lead to fixed and irreversible airway obstruction. It has been demonstrated that the innate and adaptive immunity system can interact with airway structural cells to promote the progression of airway remodeling in synergy [10]. Recently, increasingly more evidence indicates that airway remodeling can occur early in life, even before the detection of any inflammation, which suggests that airway remodeling is not simply a result of inflammation [11]. The direct binding of IgE to airway smooth muscle cells as well as air pollution and epigenetic events can also induce remodeling [11, 12].

## Overview of the sirtuin family

**Fig. 1:**
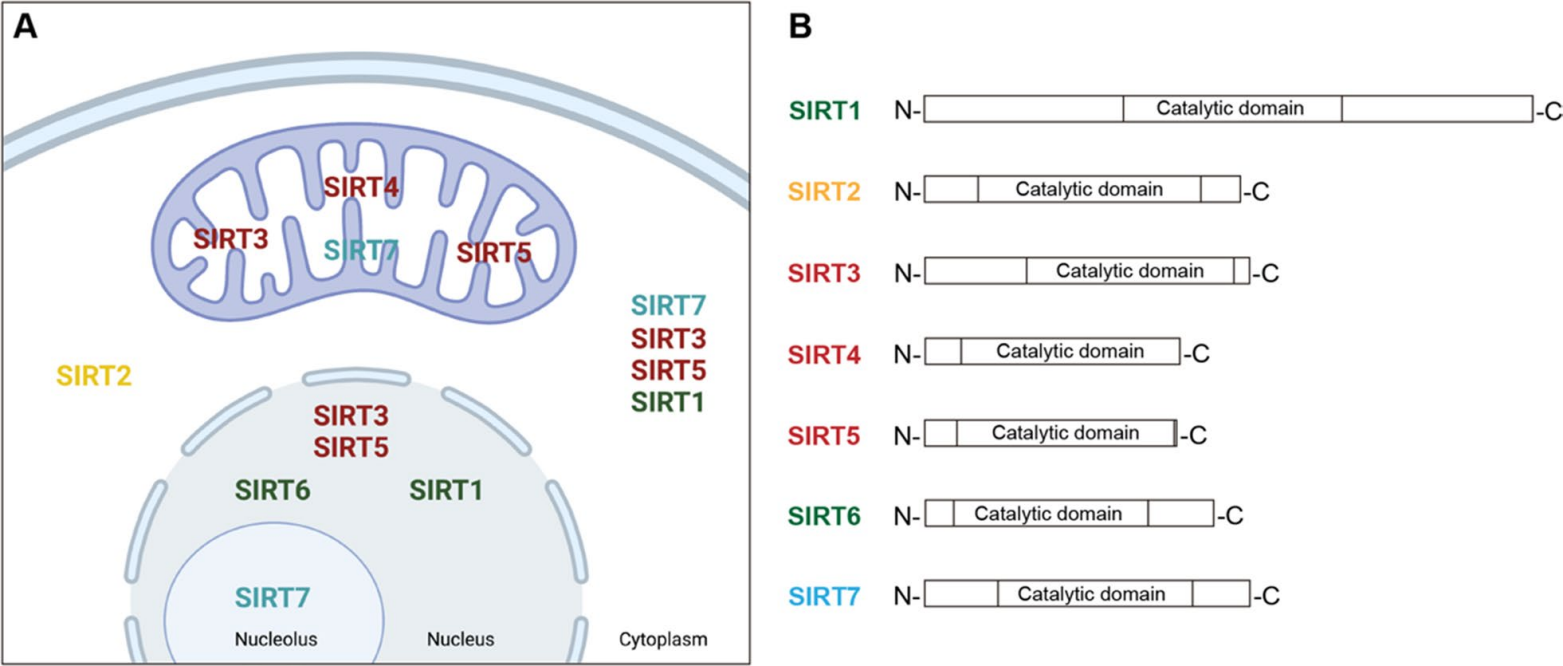
Localization (**A**) and structure (**B**) of human sirtuins

SIRTs were first identified in yeast as an important suppressor of DNA damage and a regulator of histone deacetylation [4]. To date, seven human SIRTs with distinct cellular location and function have been identified. SIRT1, SIRT6, and SIRT7 are mainly distributed in the nucleus, SIRT3, SIRT4, and SIRT5 in the mitochondria, and SIRT2 is predominantly expressed in the cytoplasm (Fig. 1) [13]. Studies have found that the expression of SIRTs is tissue-specific and cell-specific. For example, SIRT1 can be expressed in the nucleus, cytoplasm, or both, depending on different tissues [14, 15]. SIRT2 is widely expressed, and the specific localization may be related to different splice variants [13]. Although SIRT3 is mainly expressed in mitochondria, some studies have shown that SIRT3 is also expressed in the cytoplasm and nucleus [16, 17]. Similarly, SIRT5 is also expressed in the cytosol, peroxisomes, and nucleus [18]. SIRT7 expression has been detected in the cytoplasm, nucleolus, and mitochondria [19–21].

Although SIRTs differ in their localization, they are highly conserved in structure, share a nicotinamide adenine dinucleotide (NAD^+^)-binding and catalytic core domain, and exert various enzymatic and biological functions (Fig. 1) [4]. Despite the initial recognition that SIRTs primarily play a role in deacetylation, which requires NAD^+^ as a cofactor, a growing body of experiments have demonstrated that SIRTs can also act on many histone and non-histone targets through multiple deacylase and mono-ADP-ribosyltransferase activities, thereby participating in multiple pathophysiological processes, such as cardiovascular diseases, metabolic diseases, autoimmune diseases, infectious diseases, and respiratory diseases [4, 5, 22–28]. Notably, each SIRT has specific substrates and exerts distinct effects in respiratory diseases.

## Roles of sirtuins in asthma

### SIRT1

Compared with other SIRTs, more research has been done on SIRT1 and the relationship between SIRT1 and asthma [29, 30]. Among all SIRTs, SIRT1 is the protein with the longest amino acid sequence and the largest molecular weight (Fig. 1 and Table 1). The nuclear localization signal (KRKKRK) of SIRT1 localized in 41–46 residues, and the NH2-terminal region of SIRT1 contains the nuclear import and export sequences, which regulate the nucleocytoplasmic shuttling of SIRT1 [31]. Accumulating evidence indicates that SIRT1 has a wide range of targets, including histone and non-histone substrates. Therefore, SIRT1 participates in the regulation of multiple physiological functions, including DNA repair, inflammatory response, immune response, metabolism, aging, autophagy, and cancer [31–34]. The role of SIRT1 in asthma is still controversial. Most studies suggest that SIRT1 plays a protective role in asthma.

**Table 1:** Enzymatic activity and functions of sirtuins

Sirtuin	Enzymatic functions	Biological functions in asthma
SIRT1	Deacetylase	Both protective and deleterious roles in asthma
SIRT2	Deacetylase	Pro-inflammatory role
SIRT3	Deacetylase	Protective role in asthma
SIRT4	Mono-ADP-ribosylase Lipoamidase Deacylase	/
SIRT5	Deacetylase Deglutarylase Demalonylase Desuccinylase	/
SIRT6	Deacetylase defatty-acylase Mono-ADP-ribosylase	Protective role in asthma
SIRT7	Deacetylase Desuccinylase Deglutarylase	Promotes proliferation and migration of airway smooth muscle cells

Colley et al. found that the deacetylase activity and expression of SIRT1 were decreased in peripheral blood mononuclear cells (PBMCs) of patients with severe asthma [35]. The decreased activity of SIRT1 was positively correlated with decreased forced expiratory volume in 1 s and negatively correlated with IL-4 transcripts in PBMCs and total serum IgE [35]. In vitro studies using human cells indicated that inhibition of SIRT1 promoted a Th2-like phenotype in T cells [35]. Mechanistically, Colley et al. identified that SIRT1 could reduce the activity of GATA3 and its binding to response elements via deacetylating GATA3, thus decreasing the expression of IL-4 and the differentiation of Th2 cells [35]. Studies by Zhang et al. and Wang et al. showed that the expression of SIRT1 in the serum of patients with asthma was increased and was negatively correlated with pulmonary function [36, 37]. Tsilogianni et al. detected the expression of serum SIRT1 via western blot and found no correlations between serum SIRT1 levels and lung function [38]. Furthermore, their study identified no difference in serum SIRT1 levels between patients with mild to moderate asthma and those with severe asthma, and the levels of serum SIRT1 did not differ between patients receiving oral corticosteroids or biologics and those not receiving oral corticosteroids or biologics [38]. Considering the protein instability of SIRT1, different levels of serum SIRT1 at different ages, and the susceptibility to various factors affecting serum, more rigorous experiments that include more asthma patients are needed to further determine the feasibility of SIRT1 serum level as a biomarker of asthma. Zhang et al. collected bronchial epithelial cells using bronchoscopic brushing from patients with severe asthma who were undergoing inhaled corticosteroid therapy. They found that the mRNA level of SIRT1 decreased in patients with asthma [39]. Studies on animal models of asthma have shown that the expression of SIRT1 is downregulated in the lung tissues and upregulated in serum [37, 40]. Administration of SRT1720, a SIRT1 activator, could suppress airway inflammation in ovalbumin (OVA)-induced mouse models of asthma [40].

As a natural polyphenolic compound with antioxidant and anti-inflammatory properties, resveratrol also exerts anti-asthmatic effects [41, 42]. The study results of Tang et al. indicated that SIRT1 attenuated airway inflammation by decreasing serine/threonine-protein kinase (Akt) phosphorylation and IL-6 expression [41]. Chen et al. demonstrated that resveratrol played a role by upregulating the expression of phosphatase and tensin homolog (PTEN) via activating SIRT1 [42]. Both gentiopicroside and pterostilbene have anti-inflammatory roles in asthma via upregulating the expression of SIRT1 [43, 44]. Additionally, myricetin increases the deacetylase activity of SIRT1, thus inhibiting tumor necrosis factor α (TNF-α)-induced nuclear factor kappa-light-chain-enhancer of activated B cells (NF-κB) activation and reducing the inflammatory response [45]. Moreover, bergenin can inhibit the TNF-α-induced pro-inflammatory response by enhancing SIRT1 activity but not SIRT1 expression, thereby blocking the NF-κB signaling pathway, ultimately leading to decreased expression of IL-6 and IL-8 [46].

Lai et al. found that *Sirt1*^*fl/fl*^*-LysMcre* mice developed enhanced airway inflammation and mucus production in response to treatment for house dust mite (HDM), which suggests that activation of SIRT1 in macrophages has an anti-inflammatory role in asthma [47]. Particulate matter (PM) is closely associated with an increased incidence of asthma and frequent exacerbations. Tien et al. and Shan et al. found that adverse health issues caused by PM may act through SIRT1, and SIRT1 functions as a protector in this process [48, 49]. MiR-138-5p targets SIRT1, thus leading to downregulation of SIRT1 [50, 51]. Inhibition of miR-138-5p can alleviate allergic symptoms and airway inflammation via upregulation of SIRT1 [50, 51].

Interestingly, in vitro and in vivo studies suggest that lung function is associated with circadian rhythms, and circadian rhythms regulate the pathophysiology of various chronic lung diseases, including asthma [52–54]. Clinical bronchial brushing specimens reveal that clock gene expression is downregulated in patients with asthma compared with controls [55]. SIRT1 participates in regulating circadian rhythms through post-translational modification of circadian clock proteins, such as brain and muscle aryl hydrocarbon receptor nuclear translocator-like 1 (BMAL1) and period 2 [53, 54]. Hence, an increase in SIRT1 activity or protein expression in the lung may have beneficial effects against inflammation and circadian disruption in the pathogenesis of asthma. It is worth mentioning that many studies have shown that SIRT1 is involved in virus-induced asthma exacerbations [30]. Activation of SIRT1 promotes the activation of various transcription factors and viral proteins through multiple signaling pathways, which results in a decrease in pro-inflammatory cytokines and chemokines associated with inflammatory cells [30]. Finally, this may lead to the prevention or alleviation of virus-induced asthma exacerbations.

In contrast, other studies have come to a contradictory conclusion that SIRT1 plays a role in promoting airway inflammation in asthma. Kim et al. found that both nuclear expression and deacetylase activity in mouse lung tissues are increased after OVA treatment [56]. The administration of sirtinol, a SIRT1 inhibitor, alleviated airway inflammation and airway hyperresponsiveness induced by OVA [56]. Legutko et al. demonstrated that SIRT1 promoted Th2 responses and allergic airway inflammation through regulating lung DC function [57]. Moreover, administration of EX-527, a specific SIRT1 inhibitor, alleviated airway inflammation in an OVA-induced mouse model of asthma via suppressing autophagy, which also suggests the pro-inflammatory role of SIRT1 [58].

### SIRT2

Due to alternative splicing, there are three human SIRT2 isoforms in both humans and mice, namely, isoforms 1, 2, and 5 (3 in murine) [59, 60]. The expression of different isoforms is tissue-specific [13]. Increasing evidence has revealed new substrates and additional detailed functions of SIRT2. It has been demonstrated that SIRT2 participates in a wide range of biological processes, such as neurodegenerative disease, cancers, cardiac hypertrophy, and inflammatory-related diseases [61, 62]. Lee et al. demonstrated that Sirt2 isoform 3/5 was an IL-4-regulated protein in murine macrophages, which could aggravate allergic airway inflammation [60]. AGK2, a selective SIRT2 inhibitor, alleviated allergic inflammation by regulating the macrophage activation phenotype [60]. Later, it was found that miR-451 contributed to the pro-asthmatic macrophage inflammatory phenotype by decreasing the expression of SIRT2, thus participating in allergic asthmatic inflammation [63]. Kim et al. demonstrated that AGK2 could inhibit mast cell-mediated allergic airway inflammation, which also suggests that SIRT2 plays a role in mast cell-mediated airway inflammatory disease [64]. Recently, accumulating evidence has indicated that SIRT2 is involved in mitosis regulation, genome integrity, cell differentiation, cell homeostasis, aging, infection, and inflammation [61]. Whether SIRT2 can participate in the occurrence and progression of asthma through the above cellular functions remains to be studied.

### SIRT3

SIRT3 mainly localizes in mitochondria and regulates mitochondrial energy metabolism and function, thus participating in age-related diseases, cancer, heart disease, and metabolic diseases [65, 66]. Human full-length SIRT3 is localized in mitochondria, whereas short isoforms can be detected in the mitochondria, nucleus, and cytoplasm [65]. It has been demonstrated that SIRT3 plays a dual role in lung cancer. SIRT3 can inhibit cell proliferation of lung adenocarcinoma by eliminating reactive oxygen species (ROS) [67]. On the contrary, SIRT3 was also found to promote lung cancer progression by promoting the carcinogenesis of nicotinamide mononucleotide adenylyltransferase 2 and the degradation of p53 [66]. Additionally, SIRT3 plays a protective role in acute lung injury through alleviating inflammation and increasing manganese superoxide dismutase-mediated antioxidation [68, 69]. SIRT3 has been shown to be involved in chronic obstructive pulmonary disease (COPD) and PM-induced impairment of airway epithelial cell function [70, 71]. Song et al. found that Sirt3 expression was decreased in asthmatic mice, and overexpression of Sirt3 could reduce bronchial epithelial cell apoptosis, oxidative stress, and inflammation in bronchoalveolar lavage fluid which means that SIRT3 is involved in asthma and has the potential to be a target for the treatment of asthma [72]. This is the only searchable study about the function of SIRT3 in asthma to date. Further research is needed on the relationship between SIRT3 and asthma.

### SIRT4 and SIRT5

Less research has been conducted on SIRT4 and SIRT5 compared with other SIRTs. However, interest in SIRT4 and SIRT5 has recently increased considerably, which has led to the identification of a large number of mechanisms, a considerable number of new substrates, and SIRT4- and SIRT5-related proteins. SIRT4 is exclusively expressed in mitochondria, and many substrates of SIRT4 are involved in mitochondrial metabolism, such as glutamate dehydrogenase and ADP or adenosine triphosphate (ATP) carrier proteins [73]. In addition, SIRT4 is also involved in lipid homeostasis, amino acid metabolism, oxidative stress, insulin secretion, and mitochondrial-localized tumor suppression [73, 74]. The deacetylase activity of SIRT5 is extremely weak, and under basal and unstressed conditions, no obvious abnormal phenotype and metabolic abnormalities were observed in *Sirt5*-null mice [75, 76]. Studies have demonstrated that SIRT5 plays an important role in catalyzing the removal of some types of negatively charged lysine acyl modifications, such as succinyl, malonyl, and glutaryl groups; as in the absence of *Sirt5*, the succinylation, malonylation, and glutarylation of a large number of mitochondrial, cytoplasmic, and nuclear proteins are increased [77]. There are four isoforms of human SIRT5 because of alternative splicing and only one isoform of murine Sirt5, which corresponds to human SIRT5 isoform 1 [78]. SIRT5 polymorphisms may be correlated with lifespan in humans [77]. It is now known that SIRT5 participates in regulating various processes, such as glycolysis, fatty acid oxidation, electron transport chain, ketone body formation, and the ricarboxylic acid cycle, thus playing pivotal roles in maintaining metabolic and cellular homeostasis. Like SIRT3, studies have shown that SIRT5 can play a tumor-promoting or tumor-suppressing role [77]. Hershberger et al., Liu et al., Gao et al., Koronowski et al., and Sadhukhan et al. indicated that SIRT5 serves a protective function in cardiovascular disease and neurodegenerative disease [79–83]. These results are an extension of our understanding of the role of SIRT4 and SIRT5 in physiological and pathophysiological conditions, thereby providing strong evidence for SIRT4 and SIRT5 as potential therapeutic targets for different diseases. However, the roles of SIRT4 and SIRT5 in the pathogenesis of asthma are not yet clear.

### SIRT6

SIRT6 has emerged as a critical player in the onset and progression of many human diseases, including lifespan regulation, cardiovascular diseases, neurodegenerative diseases, viral infections, cancer, diabetes, and inflammation, functioning via its enzymatic activity [84]. Of these, the most direct evidence is that SIRT6-deficient mice develop aging-related degenerative phenotypes, genomic instability, and DNA damage hypersensitivity [85]. Cancer-related studies suggest that SIRT6 is a double-edged sword. SIRT6 may act as both an oncogene and an anti-oncogene in non-small cell lung cancer (NSCLC) [84, 86]. SIRT6 may also play a certain protective role in COPD and its extrapulmonary symptoms [24]. Jiang et al. found that Sirt6 is upregulated in asthmatic mice, and overexpression of Sirt6 significantly attenuates OVA- and HDM-induced airway inflammation and airway hyperresponsiveness to methacholine [87]. Mechanistically, it was demonstrated that Sirt6 deacetylates GATA3, thereby leading to negative regulation of type 2 responses induced by OVA or HDM [87]. Liu et al. investigated the expression of SIRT6 and airway remodeling in an asthma model. Their results indicated that SIRT6 expression was upregulated during airway remodeling and that SIRT6 modulated the epithelial–mesenchymal transition (EMT) in bronchial epithelial cells via the mothers against decapentaplegic homolog 3 (Smad3)-c-Jun pathway [88]. Both studies suggest that SIRT6 may serve as a new target for the treatment of asthma.

### SIRT7

SIRT7 is the only mammalian SIRT that principally resides in the nucleolus. Although SIRT7 can exhibit deacetylase, desuccinylase, and deglutarylase activities, there are SIRT7 cellular functions independent of its enzymatic activity [89, 90]. It has been demonstrated that SIRT7 has a critical role in diverse cellular processes, ranging from gene regulation to genome stability, ageing, tumorigenesis, endoplasmatic reticulum stress, mitochondrial protein folding stress, and mitochondrial metabolism [91]. Much support has come from the use of *Sirt7* knockout mice [92, 93]. Lung cancer-related studies show that SIRT7 is overexpressed in NSCLC and SIRT7 can promote human NSCLC cell growth and metastasis via regulating G1-to-S-phase transition, EMT, and activation of Akt and extracellular signal-regulated kinase 1/2 (ERK1/2) signaling [94, 95]. Additionally, the expression of SIRT7 is associated with anti-metabolic therapy in NSCLC cells [96]. There are no reports on the role of SIRT7 in COPD. As for asthma, Fang et al. found that the expression of SIRT7 was increased in airway smooth muscle (ASM) cells treated with transforming growth factor-beta 1 (TGF-β1), and SIRT7 participated in regulating TGF-β1-induced ASM cell proliferation and migration by regulating TGF-β receptor I (TβRI), suggesting the important role of SIRT7 in asthmatic airway remodeling [97]. Further research on the role of SIRT7 in asthma is required to make a conclusive claim.

## Concluding remarks and future perspectives

Owing to the elucidation of SIRT structure, enzymatic activities, and biological functions, huge progress has been made in the development of their modulators, including inhibitors, activators, and other natural products or analogs that can modulate SIRTs. Accumulating evidence demonstrates that SIRTs are involved in various pathophysiological processes, which suggests that SIRTs have the potential to be therapeutic targets for many diseases. As for the practical application of SIRT modulators in the treatment of asthma, there is still a long way to go. Compared with other SIRTs, more studies have been conducted on the role of SIRT1 and its modulators in asthma. We summarize related studies published on SIRT1 in Table 2.

**Table 2:** Involved mechanisms or effects of modulators of SIRT1 in asthma

Modulators	Models or specimens	Involved mechanisms or effects
*Activators*		
SRT1720 [38]	OVA-induced asthma mouse model	Decrease total and eosinophil cell counts and IL-5 and IL-13 levels in the BAL fluid, alleviate inflammatory cell lung infiltrates histologically, suppress cell proliferation, decrease IL-6 and TNF-α production in splenocytes
Resveratrol [38]	OVA-induced asthma mouse model	Suppress cell proliferation, decrease IL-6 and TNF-α production in splenocytes
Resveratrol [40]	OVA-induced asthma mouse model	Alleviate OVA-induced airway inflammation and airway remodeling via PTEN pathway
Gentiopicroside [41]	OVA-induced asthma mouse model	Alleviate airway inflammation through regulating SIRT1/NF-κB p65 signaling pathway
*Inhibitors*		
Sirtinol [33]	PBMCs from healthy subjects, CD4 + CD45RA + T cells from human cord blood, and HUT78 T lymphocytes	Increase the expression of Th2-associated cytokines, and increase acetylation of GATA-3 protein
Salermide [39]	human airway epithelial cell line 16HBE, and OVA-induced asthma mouse model	Increased IL-6 mRNA and protein levels via activation of Akt
Sirtinol [54]	OVA-induced asthma mouse model	Attenuate airway inflammation and hyperresponsiveness through the modulation of vascular endothelial growth factor expression mediated by HIF-1α
Cambinol [55]	OVA-induced asthma mouse model	Alleviate airway inflammation and hyperresponsiveness, decrease Th2 cytokine production and T cell proliferation in DCs through the derepression of PPAR-γ
Sirtinol [55]	OVA-induced asthma mouse model	Alleviate airway inflammation and hyperresponsiveness, decrease Th2 cytokine production and T cell proliferation in DCs through the derepression of PPAR-γ
EX-527 [56]	OVA-induced asthma mouse model	Reduce airway inflammation through the mTOR-mediated autophagy pathway

OVA: ovalbumin; IL-5: interleukin 5; BAL: bronchoalveolar lavage; TNF-α: tumor necrosis factor-α; PTEN: phosphatase and tensin homologue; NF-κB: Nuclear factor kappa-light-chain-enhancer of activated B cells; PBMCs: peripheral blood mononuclear cells; Akt: serine/threonine-protein kinase; HIF-1α: hypoxia-inducible factor 1α; DCs: dendric cells; PPAR: peroxisome proliferator-activated receptors; mTOR: mammalian target of rapamycin

Despite advancements, research progress and clinical application of SIRT modulators have not gone smoothly. The low selectivity of SIRT modulators has become one of the main obstacles limiting research progress. As the first discovered SIRT1 activator, resveratrol can play a certain role in other SIRTs besides acting on SIRT1 [28]. Activators may have better selectivity and fewer adverse effects than inhibitors [28]. However, activators, especially specific and selective activators, are rare in number relative to inhibitors. Therefore, further study of the SIRT family mechanisms involved in the pathogenesis of asthma is needed. Moreover, we must explore and confirm the efficacy and safety of SIRT modulators in the treatment of asthma to obtain better efficacy and avoid adverse reactions.

## Data Availability

Not applicable.

## Abbreviations

SIRT: Sirtuin
Sir2: Silent information regulator 2
KDACs: Lysine deacetylases
ADP: Adenosine diphosphate
Th2: T helper 2
IL-33: Interleukin 33
DCs: Dendritic cells
IgE: Immunoglobulin E
NAD: Nicotinamide adenine dinucleotide
PBMCs: Peripheral blood mononuclear cells
OVA: Ovalbumin
Akt: Serine/threonine-protein kinase
PTEN: Phosphatase and tensin homolog
NF-κB: Nuclear factor kappa-light-chain-enhancer of activated B cells
HDM: House dust mite
PM: Particulate matter
BMAL1: Brain and muscle aryl hydrocarbon receptor nuclear translocator-like 1
ROS: Reactive oxygen species
COPD: Chronic obstructive pulmonary disease
ATP: Adenosine triphosphate
NSCLC: Non-small cell lung cancer
EMT: Epithelial-mesenchymal transition
Smad3: Mothers against decapentaplegic homolog 3
ASM: Airway smooth muscle
ERK1/2: Extracellular signal-regulated kinase 1/2
TGF-β1: Transforming growth factor-beta 1
TβRI: TGF-β receptor I
